# What could we learn from SARS when facing the mental health issues related to the COVID-19 outbreak? A nationwide cohort study in Taiwan

**DOI:** 10.1038/s41398-020-01021-y

**Published:** 2020-10-06

**Authors:** Nian-Sheng Tzeng, Chi-Hsiang Chung, Chuan-Chia Chang, Hsin-An Chang, Yu-Chen Kao, Shan-Yueh Chang, Wu-Chien Chien

**Affiliations:** 1 Department of Psychiatry, Tri-Service General Hospital, School of Medicine, National Defense Medical Center, Taipei, Taiwan; 2 Student Counseling Center, National Defense Medical Center, Taipei, Taiwan; 3 Department of Medical Research, Tri-Service General Hospital, National Defense Medical Center, Taipei, Taiwan; 4 School of Public Health, National Defense Medical Center, Taipei, Taiwan; 5 Taiwanese Injury Prevention and Safety Promotion Association, Taipei, Taiwan; 6 Department of Psychiatry, Tri-Service General Hospital, Song-Shan Branch, National Defense Medical Center, Taipei, Taiwan; 7 Division of Pulmonary and Critical Medicine, Department of Medicine, Tri-Service General Hospital, School of Medicine, National Defense Medical Center, Taipei, Taiwan; 8 Graduate Institute of Medical Sciences, National Defense Medical Center, Taipei, Taiwan; 9 Graduate Institute of Life Sciences, National Defense Medical Center, Taipei, Taiwan

**Keywords:** Psychiatric disorders, Neuroscience

## Abstract

There were several studies about the psychiatric and mental health issues related to the severe adult respiratory syndrome (SARS) outbreak in 2003, however, the association between SARS and the overall risk of psychiatric disorders and suicides has, as yet, to be studied in Taiwan. The aim of this study is to examine as to whether SARS is associated with the risk of psychiatric disorders and suicide. A total of 285 patients with SARS and 2850 controls without SARS (1:10) matched for sex, age, insurance premium, comorbidities, residential regions, level of medical care, and index date were selected between February 25 and June 15, 2003 from the Inpatient Database Taiwan’s National Health Insurance Research Database. During the 12-year follow-up, in which 79 in the SARS cohort and 340 in the control group developed psychiatric disorders or suicide (4047.41 vs. 1535.32 per 100,000 person-years). Fine and Gray’s survival analysis revealed that the SARS cohort was associated with an increased risk of psychiatric disorders and suicide, and the adjusted subdistribution HR (sHR) was 2.805 (95% CI: 2.182–3.605, *p* < 0.001) for psychiatric disorders and suicide. The SARS cohort was associated with anxiety, depression, sleep disorders, posttraumatic stress disorder/acute stress disorder (PTSD/ASD), and suicide. The sensitivity analysis revealed that the SARS group was associated with anxiety, depression, sleep disorders, PTSD/ASD, and suicide after the individuals with a diagnosis of psychiatric disorders and suicide were excluded within the first year, and with anxiety, depression, and sleep disorders, while those in the first five years were excluded. In conclusion, SARS was associated with the increased risk of psychiatric disorders and suicide.

## Introduction

The coronavirus disease 2019 (COVID-19) outbreak not only causes deaths and adverse consequences on the physical health^1,2^, but also induces a global mental health crisis, including psychiatric morbidity and suicide, in the patients, the health care professionals, and the general population^3–5^. In the meantime, in May 2020, the COVID-19 pandemic seems not, as yet, to be ameliorated^6^. It is too early to reach a conclusion about the overall impact on the mental health in the country or global levels from the limited empirical data. There are several differences between COVID-19 and severe adult respiratory syndrome (SARS) in the death rates and range of transmission rates^1^, for example, in regard of the case numbers, SARS was just not comparable to COVID-19. Furthermore, the complexities of COVID-19, such as variety of symptoms, multiple organ involvements have not been seen in SARS. On the other hand, the severe sequela in SARS patients, especially the lung fibrosis, may be not common in COVID-19^6,7^. However, they are similar in several ways: First, they were caused by two similar, but different, coronaviruses. Second, the infections have caused the large-scale influences in society^8,9^. Since the understanding of the mental health issues are urgent, we believe that we could learn some experiences from the SARS-related mental health issues, including the psychiatric disorders and suicide.

An outbreak of SARS caused by a novel coronavirus severely affected Taiwan in 2003^10^, and the first confirmed diagnosis of SARS was on February 25, 2003, and the last diagnosis on June 15, 2003. Through this endemic outbreak, there were 346 patients diagnosed with SARS and 37 died among these patients^7^ and there were also SARS outbreaks in China, Singapore, and Toronto, Canada, at about the same time^7^. There were several studies about the psychiatric and mental health issues related to this SARS outbreak. One report from Taiwan of about 10 cases has found that most of the psychiatric diagnoses in the consultation services were adjustments disorder, organic hallucinosis, and organic manic disorder during the acute phase treatment^11^. In a Hong Kong study, the post-SARS cumulative incidence of psychiatric disorders was 58.9% in a cohort with 90 patients, and the prevalence of psychiatric disorders at 30 months after the SARS was 33.3%, in which one-fourth of the patients had post-traumatic stress disorder (PTSD), and 15.6% had a depressive disorder^12^. One meta-analysis, combining the studies about SARS, Middle East Respiratory Syndrome (MERS), and COVID-19, has indicated that, in the post-illness stage, the point prevalence of PTSD was 32.2% (95% Confidence Interval [CI]: 23.7–42.0), that of depression was 14·9% (95% CI: 12.1–18.2), and that of anxiety disorders was 14.8% (95% CI 11.1–19.4)^13^. Other studies about SARS-related mental health issues were conducted in the doctors^14^, nurses^14–16^, and overall hospital workers^17^. In addition, even though several studies have warned of a potential rise of suicides in the COVID-19^18,19^, there were limited reports on the topic of post-SARS suicides^20,21^. Therefore, a nationwide, population-based, long-term study on the topic for psychiatric disorders and suicides for the patients with SARS is yet to be conducted.

Apart from the psychosocial stressors related to SARS or COVID-19^22,23^, the cytokine storms and other immunological factors might also contribute to the post-infection psychiatric morbidity^24^. In addition, the long-term adverse health outcomes for the SARS survivors could also be a risk factor for the psychiatric morbidity^25^. Therefore, we hypothesized that SARS is associated with an increased risk in the development of psychiatric disorders and suicide, and we conducted this nationwide, population-based, cohort study so as to investigate the association between SARS and the psychiatric disorders and suicide, using the National Health Insurance Research Database (NHIRD), a claims database retrieved from the whole population of Taiwan.

## Methods

### Data sources

The National health insurance (NHI) Program is a mandatory and universal health insurance program in Taiwan, which has been has been operative since 1995, that covered contracts with 97% of the medical providers with approximately 23 million beneficiaries, or more than 99% of the population^26^. The details of this program were documented in several previous studies^27–37^. The NHIRD contains comprehensive and detailed data regarding the total outpatients and inpatients. An Inpatient Dataset in 2000–2015 was selected from the NHIRD, with individual diagnoses coded by the International Classification of Disease, Ninth Revision, Clinical Modification (ICD-9-CM).

### Ethics

This study was conducted in accordance with the Code of Ethics of the World Medical Association (Declaration of Helsinki). The Institutional Review Board of the Tri-Service General Hospital approved this study and waived the need of individual consents since all the identification data were encrypted in the NHIRD (IRB: TSGHIRB No. B-109–14).

### Study design and sampled participants

**Fig. 1:**
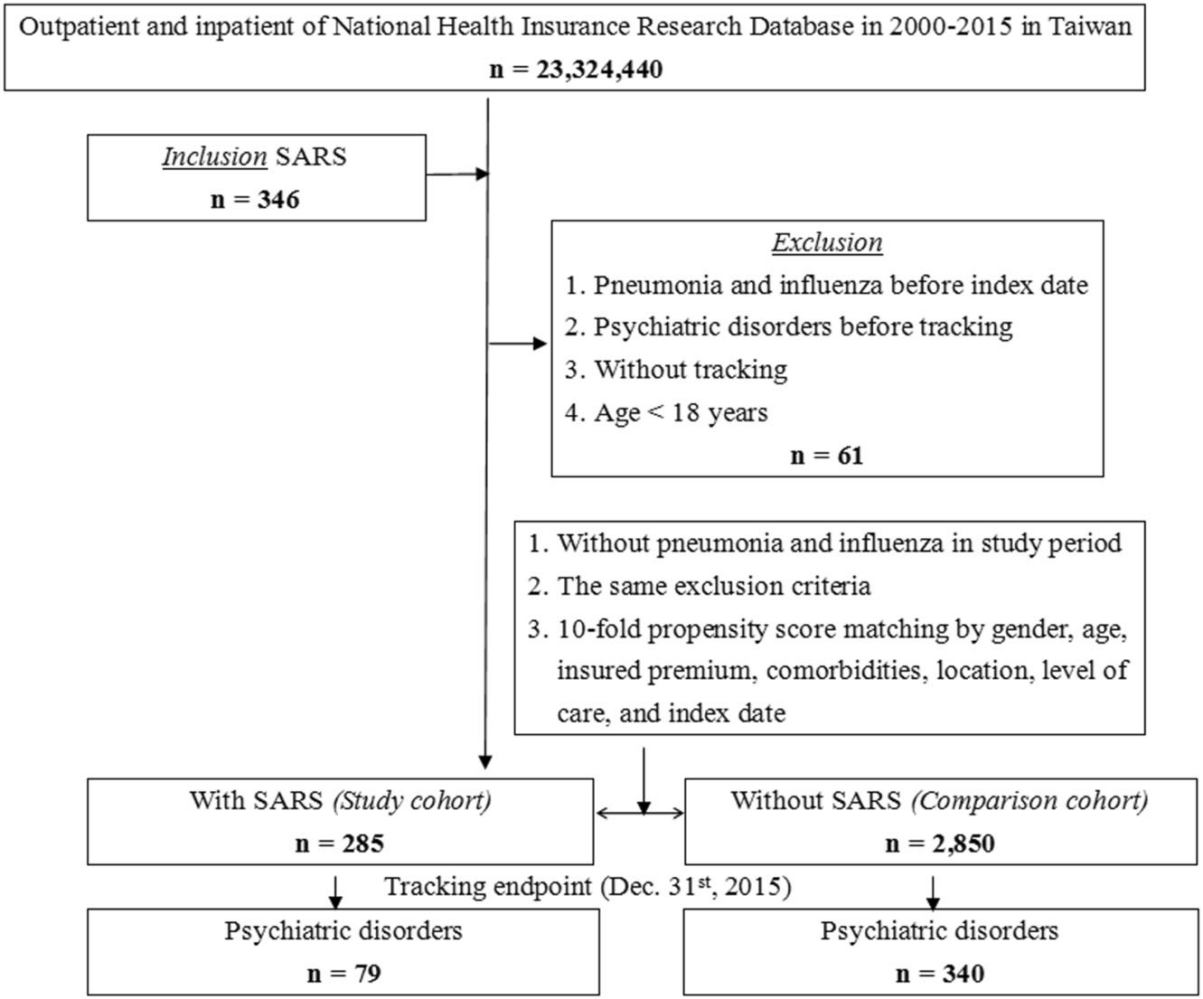
The flowchart of study sample selection. The arrows mean the selection process in the sample. The abbraviation of SARS is Severe Acute Respiratory Syndrome.

This is a retrospective matched-cohort research using the Inpatient Dataset between January 1, 2000, and December 31, 2015. Each patient with SARS was required to receive a diagnosis in an inpatient setting with the ICD codes as 480.8 and 480.9. A 1:10 sex-matched, age-matched, insurance premium-matched, comorbidities-matched, location-matched, level of care-matched, and index date-matched controls were randomly selected for each patient with SARS. The exclusion criteria for the cohorts were with unknown sex and individuals diagnosed with psychiatric disorders or pneumonia and influenza (ICD-9-CM codes: 480–488) before the index date. The index date was defined as the time when the individuals were first diagnosed as SARS within the one-year study period (Fig. 1).

### Outcomes

All of the SARS participants and controls were followed from the index date until the onset of psychiatric disorders, including anxiety disorders, depression, bipolar disorders, sleep disorders, PTSD, eating disorders, substance use related disorder, dementia, psychotic disorders, and suicide, death, withdrawal from the NHI program, or the end of 2015. The ICD codes of psychiatric disorders and suicide are as listed in Table S1. In Taiwan, the clinical diagnosis of psychiatric disorders must conform to the criteria from the Diagnostic and Statistical Manual of Mental Disorders-IV (DSM-IV), or the Diagnostic and Statistical Manual of Mental Disorders-IV (DSM-IV-TR) and confirmed by a board-certificated psychiatrist.

### Covariates

The covariates include sociodemographic characteristics and comorbidities. Sociodemographic characteristics included sex, age (18–44, 45–64, ≧65 years), education (<12 years; ≧12 years), monthly insured premiums, urbanization levels, regions of residence, and levels of medical care. The monthly insured premiums have been divided into three categories in New Taiwan Dollars [NT$]: <18,000, 18,000–34,999, ≥35,000. The urbanization level was defined by population and certain indicators of the city’s level of development. Level 1 urbanization was defined as having a population greater than 1,250,000 people. Level 2 urbanization was defined as having a population between 500,000 and 1,250,000. Urbanization levels 3 and 4 were defined as having a population between 150,000 and 500,000 and less than 150,000, respectively^38^. The Charlson comorbidity index (CCI) is one of the most widely used comorbidity indexs^39,40^, which consists of 22 conditions^41^, including myocardial infarction, congestive heart failure, peripheral vascular disease, dementia, cerebrovascular disease, chronic lung disease, connective tissue disease, ulcer, chronic liver disease, diabetes, hemiplegia, moderate or severe kidney disease, diabetes with end organ damage, tumor, leukemia, lymphoma, moderate or severe liver disease, malignant tumor, metastasis, and acquired immune deficiency syndrome (AIDS). We used the CCI to quantify the comorbidities since it could predict the in-hospital mortality or outcome in patients with severe adults respiratory infection (SARI) and other infections^42–44^.

### Statistical analysis

The SPSS software version 22 (SPSS Inc., Chicago, Illinois, USA) was used to conduct the statistical analyses. The Pearson *chi-square* test was used for the analysis of the categorical data. Continuous variables presented as the mean (±SD), were analyzed using the two-sample *t* test. To investigate the risk of psychiatric disorders and suicide for patients with and without SARS, the Fine and Gray’s model was used to conduct the competing risk analysis to calculate the subdistribution hazard ratios (sHRs) and 95% confidence intervals (CIs), adjusting for sociodemographic characteristics, and comorbidities. The Value-added module, including the Competing Risks Survival Analysis, in the SPSS was used to conduct Fine and Gray’s survival analysis (https://www.asiaanalytics.com.tw/en/product/p-asia-analytics-2.jsp). The Kaplan–Meier method was used to determine the difference in the risk of psychiatric disorders and suicide for the patients with SARS and the control cohorts using the log-rank test. A *p* value < 0.05 was considered statistically significant.

## Results

### Study cohort characteristics

Table 1 shows the sex, age, education, monthly insured premiums, urbanization levels, regions of residence, comorbidities, and levels of medical care in the patients with or without SARS. When compared to the controls, patients with SARS have had no significant difference in the covariates.

**Table 1:** Characteristics of study in the baseline.

SARS	With		Without		
Variables	*n*	%	*n*	%	*P*
Total	285	9.09	2850	90.91	
Sex					0.999
Male	106	37.19	1060	37.19	
Female	179	62.81	1790	62.81	
Age (years)	49.12 ± 12.18		49.73 ± 12.25		0.423
Age groups (years)					0.999
18–44	142	49.82	1420	49.82	
45–64	92	32.28	920	32.28	
≧65	51	17.89	510	17.89	
Insured premium (NT$)					0.479
<18,000	281	98.60	2787	97.79	
18,000–34,999	2	0.70	46	1.61	
≧35,000	2	0.70	17	0.60	
Marital status					0.384
Without	126	44.21	1339	46.98	
With	159	55.79	1511	53.02	
Education levels (years)					0.950
<12	147	51.58	1478	51.86	
≧12	138	48.42	1372	48.14	
CCI_R					0.999
0	158	55.44	1580	55.44	
1	58	20.35	580	20.35	
≧2	69	24.21	690	24.21	
Location					0.927
Northern Taiwan	132	46.32	1305	45.79	
Middle Taiwan	54	18.95	543	19.05	
Southern Taiwan	87	30.53	874	30.67	
Eastern Taiwan	11	3.86	124	4.35	
Outlets islands	1	0.35	4	0.14	
Urbanization level					0.952
1 (The highest)	154	54.04	1497	52.53	
2	69	24.21	710	24.91	
3	46	16.14	489	17.16	
4 (The lowest)	16	5.61	154	5.40	
Level of care					0.976
Hospital center	149	52.28	1485	52.11	
Regional hospital	107	37.54	1063	37.30	
Local hospital	29	10.18	302	10.60	

*SARS* severe adult respiratory syndrome, *CCI* Charlson comorbidity index, *NT$* new Taiwan dollars, *P* Chi-square/Fisher exact test on category variables and *t*-test on continue variables.

### Kaplan–Meier curves for the cumulative incidence of psychiatric disorders in patients with psychiatric disorders and suicide

**Fig. 2:**
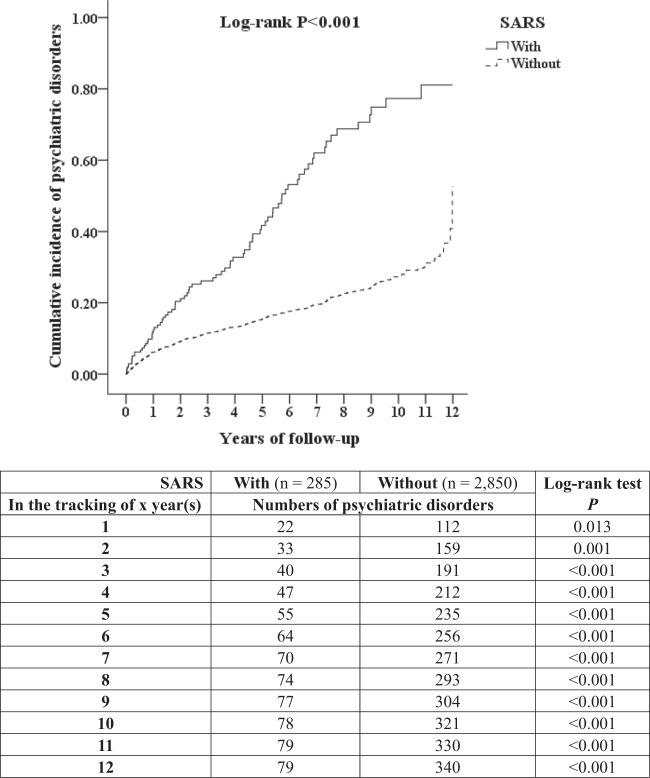
Kaplan-Meier for cumulative incidence of psychiatric disorders among aged 18 and over stratified by SARS with log-rank test. The solid line means the cumulative incidence of psychiatric disorders of the patients with SARS. The dotted line means the cumulative incidence of psychiatric disorders of the patients without SARS. The abbraviation of SARS is Severe Acute Respiratory Syndrome.

In total, 285 patients were diagnosed with SARS during the study period. During the follow-up period, 79 in the SARS group (*N* = 285) and 340 in the control group (*N* = 2850) developed psychiatric disorders or suicide (4047.41 vs. 1535.32 per 100,000 person-years). Figure 2 reveals that the difference between the two cohorts in the psychiatric disorders and suicide were significant (long-rank test, *p* < 0.001).

### Years from SARS to psychiatric disorders and suicide

The mean time from the index date to the diagnosis of psychiatric disorders and suicide after diagnosis was 3.54 (SD = 3.26) years. The mean years to the developed psychiatric disorders and suicide in patients with SARS were 3.42 (SD = 2.80) years, which was earlier than patients without SARS (3.57 [SD = 3.36] years) (Table S2).

### Subdistribution hazard ratio analysis of psychiatric disorders and suicide in the patients with SARS

Table 2 shows the factors of SARS, using Fine and Gray’s survival analysis, of the factors associated with the risk of psychiatric disorders and suicide. The crude sHR was 2.785 (95% CI: 2.136–3.527, *p* < 0.001), and after adjusting for sex, age, education, monthly insured premiums, urbanization levels, regions of residence, comorbidities, and levels of medical care, the adjusted sHR was 2.805 (95% CI: 2.182–3.605, *p* < 0.001) for psychiatric disorders and suicide. SARS patients aged 45–64 years, ≧65 years, with the CCI score ≧2, and care from the medical centers and regional hospitals, were associated with an increased risk for psychiatric disorders and suicide. Male patients with SARS were associated with a decreased risk for psychiatric disorders and suicide, in comparison to the female patients.

**Table 2:** Factors of psychiatric disorders by using Fine and Gray’s competing risk model.

	Competing risk in the model							
Variables	Crude HR	95% CI	95% CI	*P*	Adjusted sHR	95% CI	95% CI	*P*
SARS (reference: without SARS)	2.785	2.136	3.527	<0.001	2.805	2.182	3.605	<0.001
Male (reference: female)	0.674	0.520	0.773	<0.001	0.545	0.448	0.662	<0.001
Age 45–64 (reference: age 18–44)	1.699	1.489	1.906	<0.001	1.819	1.624	2.076	<0.001
Age ≧65 (reference: age 18–44)	1.825	1.671	2.038	<0.001	1.929	1.723	2.194	<0.001
CCI = 1 (reference: CCI = 0)	1.299	1.012	1.703	0.038	1.236	0.946	1.615	0.120
CCI ≧ 2 (reference: CCI = 0)	1.813	1.351	2.204	<0.001	1.882	1.672	2.158	0.001
Medical center (reference: Local hospital)	1.411	1.074	2.010	0.008	1.329	0.993	1.779	0.056
Regional hospital (reference: Local hospital)	1.209	1.034	1.903	0.017	1.184	0.917	1.529	0.195

*SARS* severe adult respiratory syndrome, *sHR* subdistribution hazard ratio, *CI* confidence interval, *CCI* Charlson comorbidity index, *Adjusted sHR* adjusted variables listed in the Table 1.

### Subgroup analysis of psychiatric disorders and suicide in the SARS cohort and controls

Table S3 shows that the SARS cohort was associated with a higher risk of psychiatric disorders than the control group, regardless of sex, age, marital status, education years, comorbidities, monthly insured premiums, residences, urbanization, and levels of care, with one exception being the insured premiums of ≧NT$ 35,000.

### Sensitivity test for analysis of the risk of psychiatric disorders and suicide in the patients with SARS

Patients with SARS were associated with an increased risk in overall psychiatric disorders, anxiety disorders, depressive disorders, sleep disorders, PTSD, and suicide, when compared to the control group. The sHR’s of these psychiatric disorders were: anxiety disorder 3.172 (95% CI:2.471–4.089, *p* < 0.001), depressive disorder 3.165 (95% CI:2.465–4.077, *p* < 0.001), sleep disorder 2.411 (95% CI:1.098–3.172, *p* = 0.001), PTSD/ASD 60.360 (95% CI:49.121–77.602, *p* < 0.001), and suicide 4.382 (95% CI:3.401–5.513, *p* < 0.001). The sensitivity analysis revealed that the SARS cohort was associated with anxiety, depression, sleep disorders, PTSD/ASD, and suicide after the individuals with a diagnosis of psychiatric disorders and suicide were excluded within the first year, and with anxiety, depression, and sleep disorders, while those in the first five years were excluded (Table 3).

**Table 3:** Factors of psychiatric disorders subgroup and sensitivity test by using Fine and Gray’s competing risk model.

	SARS	With		Without (Reference)		Competing risk in the model			
Sensitivity test	Psychiatric disorders	Events	Rate (per 10^5^ PYs)	Events	Rate (per 10^5^ PYs)	Adjusted sHR	95% CI	95% CI	*P*
Overall	Overall	79	4047.41	340	1535.32	2.805	2.182	3.605	<0.001
	Anxiety	10	512.33	38	171.59	3.172	2.471	4.089	<0.001
	Depression	21	1075.89	80	361.25	3.165	2.465	4.077	<0.001
	Sleep disorders	13	666.03	64	289.00	2.411	1.098	3.172	0.001
	PTSD/ASD	5	256.16	1	4.52	60.360	49.121	77.602	<0.001
	Suicide	4	204.93	11	49.67	4.382	3.401	5.513	<0.001
In the first year excluded	Overall	57	2995.04	228	1043.54	2.749	2.136	3.532	<0.001
	Anxiety	10	525.45	26	119.00	3.109	2.403	3.972	<0.001
	Depression	18	945.80	62	283.77	3.100	2.389	3.985	<0.001
	Sleep disorders	9	472.90	45	205.96	2.345	1.076	3.101	0.002
	PTSD/ASD	3	157.63	1	4.58	50.121	34.256	64.230	<0.001
	Suicide	2	105.09	6	27.46	4.201	3.456	4.986	<0.001
In the first 5 years excluded	Overall	24	1,567.32	105	575.14	2.392	1.858	3.072	<0.001
	Anxiety	4	261.22	11	60.25	2.703	2.094	3.454	<0.001
	Depression	9	587.74	27	147.89	2.688	2.063	3.442	<0.001
	Sleep disorders	2	130.61	20	109.55	2.035	1.404	2.682	<0.001

*SARS* severe adult respiratory syndrome, *PYs* Person-years, *Adjusted HR* adjusted subdistribution hazard ratio: adjusted for the variables listed in Table 1., *CI* confidence interval, *PTSD/ASD* post-traumatic stress disorder/acute stress disorder.

## Discussion

In this study, we have several noteworthy findings: First, the SARS cohort had a 2.8-fold increased risk of overall psychiatric disorders when compared to the control cohort. Compared with previous reports about the association between SARS and psychiatric disorders, such as a case series^11^, or a smaller sample size study^12^, this study was based on a nationwide, population-based claims database, with a larger sample size, in a long-duration follow-up. Even though one meta-analysis, combining SARS, MERS, and COVID-19 studies, has been comprised of 1991 cases of acute phase SARS^13^, this study has a longer follow-up for the risk of developing psychiatric disorders. To the best of our knowledge, this is the first study on the association between SARS and increased risk in developing psychiatric disorders and suicide, in a 12-year follow-up, from a nationwide, population-based database.

Second, we investigated the risk of different psychiatric diagnoses in the SARS patients, and we found that SARS was associated with anxiety, depression, sleep disorders, PTSD/ASD, and suicide, which is similar to the findings of the association between severe coronavirus infections and PTSD^12,13^, anxiety^13^, and depressive disorders^12,13^. However, we found that the risk of psychiatric disorders could be increased even in a long term follow-up of 12 years, not just a shorter term of up to 30-month of follow-up^12^, in this study.

Third, to resolve the influences of protopathic bias, we conducted the sensitivity analysis: the SARS cohort was associated with anxiety, depression, sleep disorders, PTSD/ASD, and suicide after the individuals with a diagnosis of psychiatric disorders and suicide were excluded within the first year, and with anxiety, depression, and sleep disorders, while those in the first five years were excluded. By conducting this analysis, we could avoid the protopathic bias, that arises when the initiation of the exposure occurs in response to an undiagnosed disease under study outcome^45^.

Fourth, in this study, we could also point out that the risk of development of PSTD was not beyond the first five years. In addition, the mean years to developed psychiatric disorders and suicide in patients with SARS were 3.42 (SD = 2.80) years. Therefore, this finding could serve as an important reminder for the clinicians caring the SARS survivors in monitoring their mental conditions in the first 3–4 years, especially for PTSD/ASD. Furthermore, we found that the SARS cohort was at a particularly high risk of PTSD/ASD: the SARS cohort had a 60-fold increased risk of PTSD/ASD and a 50-fold increased risk of PTSD/ASD even after excluding the psychiatric disorders within the first year. This finding is similar to several studies on the topic of the association between severe coronavirus infections and PTSD, up to one-third to one-fourth of the patients of the severe coronavirus infections^12,13^.

Fifth, suicide after the SARS outbreak was a critical issue, and previous studies on this topic have investigated the potential increased suicide risk in the elderly^20,46^ and the ED visitors^21^, during and after the SARS outbreak. This study might be the first study about the suicides of the SARS survivors. As aforementioned, the association between SARS and suicide was statistically significant even after excluding suicide within the first year, but not significant after excluding suicide within the first five years. Therefore, the careful evaluation in the suicide risk is important in the following years after SARS.

The underlying mechanisms for the risk of psychiatric disorders remained unclear. One of the possible reasons for the increased risk of psychiatric disorders and suicide might well be related to the psychological impact for the patients, general stress, and the negative psychological effects are increased in the SARS patients, particularly among the infected health care workers^47,48^, such as stress from the quarantine and isolation, fear of uncertainty and death, impaired health after severe viral infections, economic burden after SARS, and even negative feelings for the SARS-related information^48–50^.

Furthermore, one previous study found that the elevated levels of the monocyte chemoattractant protein-1 (MCP-1), transforming growth factor beta-1 (TGFβ-1), interleukin-1 beta (IL-1 β), and interleukin-6 (IL-6) in the SARS-CoV-infected angiotensin-converting enzyme 2 (ACE2+) Cells in the SARS patients^51^. The increased levels of these cytokine might not only result in acute lung injury, but also be associated with psychiatric disorders, such as depressive disorders, bipolar, or anxiety disorders^52,53^. In this study, patients aged 45–64 years or ≧65 years, and a higher CCI score of ≧2, and the level of care from the medical centers and regional hospitals, were associated with the risk of psychiatric disorders and suicide. This finding hints that patients with older ages, and more severe physical morbidities in the SARS patients, could also contribute to the risk of psychiatric disorders. Nonetheless, further studies are needed to investigate the underlying mechanisms for the development of psychiatric disorders in the SARS survivors.

### Strengths of this study

This study has several strengths: First, we used the LHID, which has a large sample size in this study. Second, SARS was diagnosed by the serology test and being a notifiable disease it needed to be reported to the Health authority in Taiwan (https://www.cdc.gov.tw/En), Third, we have conducted a long-term follow-up of 12 years of the development of psychiatric disorders after the SARS outbreak in 2003. This could serve as a lesson for us to learn from SARS when facing the challenge of psychiatric disorders from COVID-19: The mental health issues in COVID-19 might not be only from the acute-phase of delirium^54,55^, depression, and acute trauma-related psychiatric disorders such as ASD or acute PTSD^56^. A long-term follow-up of psychiatric disorders for COVID-19 survivors would be important.

### Limitations of this study

This study has several limitations that warrant consideration. First, similar to previous studies using the NHIRD on infectious, parasitic, or inflammatory diseases^57–61^, since the severity, weakness severity, laboratory parameters, or lung function examinations in SARS patients were not recorded in the NHIRD. Second, other factors, such as genetic, psychosocial, and environmental factors, were not included in the dataset. Third, ascertainment bias is possible if the patients who were treated for SARS were more medically attentive so that they also sought treatment for psychiatric conditions. Fourth, this study contained only small number of cases, and thus there are very few numbers of PTSD and suicide, which all happened within five years after SARS. Furthermore, for other psychiatric cases, nearly one third of the cases happened within one year, and up to two thirds reported with five years both in the SARS and control cohorts, only one occurred after ten years in SARS group (Table 2). Considering the new occurrence of psychiatric disorders, the difference was not so significant between two cohorts after nine years in the study period. Therefore, most of the attention should be paid to psychiatric disorders that developed within ten years of the onset of SARS. After all, the small numbers of SARS cases limit the generalization of the SARS experiences to the diagnosis and treatment of psychiatric consequences of COVID-19 treatment. Finally, the NHIRD does not contain the information for the SARS patients’ family members and the health workers who take care of them. We need further studies to investigate the post-SARS psychiatric morbidity in the long-term follow-up.

## Conclusion

This study found that SARS was associated with the increased risk of psychiatric disorders and suicide in a long-term follow-up study of 12 years. This is also a reminder for the clinicians that psychiatric morbidity is an important issue in the patients with severe coronavirus infections, such as COVID-19.

## Supplementary information

Table S1

Table S2

Table S3

## Data Availability

Data are available from the National Health Insurance Research Database (NHIRD) published by the Taiwan National Health Insurance (NHI) Administration. Due to legal restrictions imposed by the government of Taiwan in relation to the “Personal Information Protection Act”, data cannot be made publicly available. Requests for data can be sent as a formal proposal to the NHIRD (https://dep.mohw.gov.tw/dos/np-2497-113.html).
