# Supplementary material for: What could we learn from SARS when facing the mental health issues related to the COVID-19 outbreak? A nationwide cohort study in Taiwan
Source: Transl Psychiatry. 2020 Oct 6;10:339. doi: 10.1038/s41398-020-01021-y (PMC7538046; doi:10.1038/s41398-020-01021-y)
Supplement: Supplementary file 1 — Table S1 [file 41398_2020_1021_MOESM1_ESM.docx]

| **Table S1. The ICD-9-CM codes of psychiatric disorders and suicide** | |
| --- | --- |
|  | **ICD-9-CM codes** |
| anxiety disorders | 300.x |
| depressive disorders | 296.2, 296.3, 300.4, 311 |
| bipolar disorders | 296.0x, 296.4x, 296.5x, 296.6x, 296.7x, 296.8x |
| sleep disorders | 307.4x, 780.5x |
| posttraumatic stress disorder | 309.81 |
| eating disorders | 307.1, 307.5 |
| substance use related disorders | 291, 292, 303.0, 303.9, 304-305 |
| dementia | 290.0, 290.1x, 290.2x, 290.3, 290.41x, 290.8, 290.9, 331.0 |
| psychotic disorders | 295.xx, 297.xx, 298.xx |
| suicide | E950-E959 |
| **ICD-9-CM = International Classification of Disease, Ninth Revision, Clinical Modification** | |
