# Supplementary material for: What could we learn from SARS when facing the mental health issues related to the COVID-19 outbreak? A nationwide cohort study in Taiwan
Source: Transl Psychiatry. 2020 Oct 6;10:339. doi: 10.1038/s41398-020-01021-y (PMC7538046; doi:10.1038/s41398-020-01021-y)
Supplement: Supplementary file 2 — Table S2 [file 41398_2020_1021_MOESM2_ESM.docx]

| **Table S2. Years to psychiatric disorders** | | | | |
| --- | --- | --- | --- | --- |
| **SARS** | **Min** | **Median** | **Max** | **Mean ± SD** |
| **With** | 0.02 | 2.24 | 10.83 | 3.42 ± 2.80 |
| **Without** | 0.01 | 2.76 | 11.89 | 3.57 ± 3.36 |
| **Total** | 0.01 | 2.32 | 11.89 | 3.54 ± 3.26 |

**SARS = Severe Adult Respiratory Syndrome, SD= standard deviation**
