# Supplementary material for: What could we learn from SARS when facing the mental health issues related to the COVID-19 outbreak? A nationwide cohort study in Taiwan
Source: Transl Psychiatry. 2020 Oct 6;10:339. doi: 10.1038/s41398-020-01021-y (PMC7538046; doi:10.1038/s41398-020-01021-y)
Supplement: Supplementary file 3 — Table S3 [file 41398_2020_1021_MOESM3_ESM.docx]

| **Table S3. Factors of psychiatric disorders stratified by variables listed in the table by using Fine & Gray's competing risk model** | | | | | | | | | | |
| --- | --- | --- | --- | --- | --- | --- | --- | --- | --- | --- |
| **SARS** | **With** | | | **Without** *(Reference)* | | | **Competing risk in the model** | | | |
| **Stratified** | **Events** | **PYs** | **Rate (per 10^5^ PYs)** | **Events** | **PYs** | **Rate (per 10^5^ PYs)** | **Adjusted sHR** | **95% CI** | **95% CI** | ***P*** |
| **Total** | 79 | 1,951.87 | 4,047.41 | 340 | 22,145.19 | 1,535.32 | 2.805 | 2.182 | 3.605 | <0.001 |
| **Sex** |  |  |  |  |  |  |  |  |  |  |
| Male | 33 | 808.18 | 4,083.25 | 171 | 7,470.53 | 2,288.99 | 1.897 | 1.475 | 2.456 | <0.001 |
| Female | 46 | 1,143.69 | 4,022.07 | 169 | 14,674.66 | 1,151.65 | 3.715 | 2.875 | 4.781 | <0.001 |
| **Age groups (years)** |  |  |  |  |  |  |  |  |  |  |
| 18-44 | 43 | 997.72 | 4,309.82 | 134 | 7,491.18 | 1,788.77 | 2.562 | 1.994 | 3.284 | <0.001 |
| 45-64 | 21 | 489.77 | 4,287.69 | 120 | 7,368.05 | 1,628.65 | 2.800 | 2.178 | 3.587 | <0.001 |
| ≧65 | 15 | 464.37 | 3,230.17 | 86 | 7,285.97 | 1,180.35 | 2.913 | 2.265 | 3.705 | <0.001 |
| **Insured premium (NT$)** |  |  |  |  |  |  |  |  |  |  |
| <18,000 | 78 | 1,884.35 | 4,139.36 | 336 | 21,531.15 | 1,560.53 | 2.825 | 2.198 | 3.685 | <0.001 |
| 18,000-34,999 | 1 | 43.65 | 2,291.01 | 3 | 493.05 | 608.46 | 3.989 | 3.111 | 5.101 | <0.001 |
| ≧35,000 | 0 | 23.87 | 0.00 | 1 | 120.99 | 826.51 | 0.000 | - | - | 0.992 |
| **Marital status** |  |  |  |  |  |  |  |  |  |  |
| Without | 48 | 876.66 | 5,475.34 | 212 | 10,419.88 | 2,034.57 | 2.862 | 2.222 | 3.665 | <0.001 |
| With | 31 | 1,075.21 | 2,883.16 | 128 | 11,725.32 | 1,091.66 | 2.808 | 2.181 | 3.621 | <0.001 |
| **Education levels (years)** |  |  |  |  |  |  |  |  |  |  |
| <12 | 44 | 993.89 | 4,427.06 | 214 | 11,305.77 | 1,892.84 | 2.485 | 1.935 | 3.198 | <0.001 |
| ≧12 | 35 | 957.98 | 3,653.52 | 126 | 10,839.43 | 1,162.42 | 3.342 | 2.603 | 4.284 | <0.001 |
| **CCI_R** |  |  |  |  |  |  |  |  |  |  |
| 0 | 27 | 785.73 | 3,436.31 | 198 | 12,368.21 | 1,600.88 | 2.284 | 1.774 | 2.936 | <0.001 |
| 1 | 18 | 354.90 | 5,071.91 | 69 | 4,119.62 | 1,674.91 | 3.221 | 2.506 | 4.101 | <0.001 |
| ≧2 | 34 | 811.25 | 4,191.08 | 73 | 5,657.36 | 1,290.35 | 3.452 | 2.687 | 4.345 | <0.001 |
| **Urbanization level** |  |  |  |  |  |  |  |  |  |  |
| 1 (The highest) | 22 | 672.17 | 3,273.00 | 73 | 6,816.26 | 1,070.97 | 3.251 | 2.525 | 4.178 | <0.001 |
| 2 | 33 | 782.12 | 4,219.31 | 150 | 10,081.21 | 1,487.92 | 3.008 | 2.342 | 3.872 | <0.001 |
| 3 | 7 | 164.11 | 4,265.39 | 34 | 1,736.39 | 1,958.08 | 2.317 | 1.802 | 2.988 | <0.001 |
| 4 (The lowest) | 17 | 333.47 | 5,097.90 | 83 | 3,511.34 | 2,363.77 | 2.287 | 1.785 | 2.945 | <0.001 |
| **Level of care** |  |  |  |  |  |  |  |  |  |  |
| Hospital center | 24 | 620.60 | 3,867.24 | 94 | 7,331.24 | 1,282.18 | 3.201 | 2.485 | 4.123 | <0.001 |
| Regional hospital | 36 | 922.71 | 3,901.53 | 143 | 9,399.66 | 1,521.33 | 2.735 | 2.106 | 3.602 | <0.001 |
| Local hospital | 19 | 408.56 | 4,650.52 | 103 | 5,414.29 | 1,902.37 | 2.607 | 2.011 | 3.375 | <0.001 |
| **SARS = Severe Adult Respiratory Syndrome; NT$ = New Taiwan Dollars; PYs = Person-years; Adjusted sHR = Adjusted Subdistribution Hazard ratio: Adjusted for the variables listed in Table 1.; CI = confidence interval** | | | | | | | | | | |
